# Dynamics and Complexity of Computrons

**DOI:** 10.3390/e22020150

**Published:** 2020-01-27

**Authors:** Murat Erkurt

**Affiliations:** Department of Mathematics, Centre for Complexity Science, Imperial College London, South Kensington campus, London SW7 2AZ, UK; murat.erkurt09@imperial.ac.uk

**Keywords:** complexity, computron, cellular automata, general network automata, diversity measure, entanglement

## Abstract

We investigate chaoticity and complexity of a binary general network automata of finite size with external input which we call a *computron*. As a generalization of cellular automata, computrons can have non-uniform cell rules, non-regular cell connectivity and an external input. We show that any finite-state machine can be represented as a computron and develop two novel set-theoretic concepts: (i) *diversity space* as a metric space that captures similarity of configurations on a given graph and (ii) *basin complexity* as a measure of complexity of partitions of the diversity space. We use these concepts to quantify chaoticity of computrons’ dynamics and the complexity of their basins of attraction. The theory is then extended into probabilistic machines where we define fuzzy basin partitioning of recurrent classes and introduce the concept of ergodic decomposition. A case study on 1D cyclic computron is provided with both deterministic and probabilistic versions.

## 1. Introduction

Complexity of dynamical systems and that of computational machines are mostly disconnected areas of research. The dynamical systems typically live in metric state spaces and evolve in time by iterating the rules of dynamics. The system is said to be complex if the information required to describe the evolving paths is high, a notion formally defined as Kolmogorov–Sinai entropy. On the other side of the world where computational machines live, the aim is to map a set of inputs to a set of outputs. We define *computron* as a computational machine that is composed of a finite number of cells placed on vertices of a directional graph. The cells have binary states which are updated according to local transition rules as a function of connected cells’ states. Computron is the generalization of a cellular automata where local rules are not necessarily uniform, and graph not required to be a regular grid. Furthermore, a computron can have an external input, in which case it is called *driven*, otherwise *autonomous*. The computron’s global state is the configuration of its cells’ local states on its graph. It performs computation by mapping an initial configuration to an attractor, be it fixed or periodic by iterative dynamics. Such mapping partitions its configuration space.

We attempt to bridge disparate worlds of dynamical systems and computation by embedding computrons in a suitably defined metric space that captures similarity of its configurations. We develop notions of dynamical chaoticity using Lyapunov characterization of the machine’s evolution and computational complexity by analyzing the basin partitioning of this proposed metric space. Our work contributes to literature in multiple ways:

1. Computron is introduced as a computational machine which is equivalent to a finite-state machine, but has the novelty that *flow of information* and *processing of information* is separated.
2. A new distance measure is introduced for configuration space on automata graphs. It is different than Hamming distance used in the literature since it captures pattern similarity of configurations on the underlying graph as opposed to solely counting mismatch between them.
3. A new complexity measure is proposed which captures the intrinsic structural complexity of a partitioning of the configuration space, not its randomness.
4. The above concepts are applied to quantify dynamics of generalized graph automata whereas literature is mainly focused on dynamics of cellular automata.
5. The framework is expanded to probabilistic automata using fuzzy basins and recurrent classes which enables us to quantify chaoticity and complexity of probabilistic machines, as well as their susceptibility to noise.

Section 2 provides background and literature review of Lyapunov characterization and entropy of dynamical systems including cellular automata. Section 3 defines the computron model. Section 4 introduces new set-theoretic concepts such as diversity measure and entanglement. Section 5 characterizes the dynamics of computron by embedding the machine into a metric space that captures similarity of configurations. Section 6 expands into probabilistic computation where each cell performs one of many rules probabilistically, driven by a random external input. This is a major step which pulls the formal domain into Markov chains and recurrent classes. Probabilistic computation lends itself to analysis of susceptibility of computrons, including effects of noise. Proofs of Lemmas introduced in various sections are provided in the Appendix A.

## 2. Background

In his *‘A Philosophical Essay on Probabilities’* (1814), Laplace states that knowing the present and evolution laws gives full knowledge of the future. Yet, even if laws are truly known and are truly deterministic, the chaos could prevail, as described by Lorenz (1972): ‘Chaos is when the present determines the future, but the approximate present does not approximately determine the future’. The sensitivity of future to present is formalized by Lyapunov characterization. The richness of what future may bring is quantified by Kolmogorov–Sinai entropy:

### 2.1. Lyapunov Characterization

Lyapunov exponents measure the typical rate of exponential divergence of nearby trajectories in a dynamical system. Consider the following continuous-time system:

(1) $$\partial_{t}x=F\left(x\right)$$

where $x\in R^{d}$ specifies the system state, and F, a differentiable function, defines the evolution law. Let x(t) and $x^{\prime }\left(t\right)$ be two trajectories that start from close-by initial states $x^{\prime }\left(0\right)=x\left(0\right)+\delta x\left(0\right)$. The evolution of difference in trajectories, $z\left(t\right)=x^{\prime }\left(t\right)-x\left(t\right)$, can be approximated as a vector in tangent space:

(2) $$\partial_{t}z_{i}=\sum_{j=1}^{d}\partial_{x_{j}}F_{i}{|}_{x(t)}z_{j}\left(t\right)$$

and there exists an orthonormal basis $\left\{e_{i}\right\}$ such that for large *t*, the difference in trajectories can be represented as exponential growth or decay:

(3) $$z\left(t\right)=\sum_{i=1}^{d}c_{i}e_{i}e^{\lambda_{i}t}$$

The Lyapunov exponents $\left\{\lambda_{i}\right\}$ are independent of the initial state x(0) if the system is ergodic. Systems with a positive Lyapunov exponent are typically chaotic and error of prediction grows exponentially if the initial state is slightly off.

### 2.2. Kolmogorov–Sinai Entropy

If we were to discretize time and coarsen the state space into *d*-dimensional cubes of each side ϵ (indexing each cube with a symbol), then a trajectory of evolution could be approximated with a string of symbols based on the index of each cube visited at each time step. For a simple system with predictable trajectories, the information needed to represent the ensemble of such strings is less than that of a system where trajectories disperse rapidly. Let K(ϵ,t) be the total number of different strings generated by the system in *t* steps in a coarse-grained state space. The *topological entropy* of the system is defined as:

(4) $$h=\lim_{\epsilon \to 0}\lim_{t\to \infty }\frac{1}{t}K\left(\epsilon ,t\right)$$

Please note that in this definition, each different string generated by the system is counted as one. If we take into account the frequency of observing a given finite string of length T (a word $w^{T}$), and apply the Shannon entropy formulation to this stream of words, we get the information content of T-symbol paths generated by the system as:

(5) $$H^{T}=-\sum_{\left\{w^{T}\right\}}P\left(w^{T}\right)\log P\left(w^{T}\right)$$

Kolmogorov–Sinai entropy is then defined as the average information content per time step of the path, chosen as the highest one over all possible coarse graining partitions of state space {G}:

(6) $$h^{KS}=\underset{G}{\sup}\lim_{T\to \infty }\frac{1}{T}H^{T}$$

### 2.3. Basin Entropy

Dynamical systems theory has traditionally been *path-centric*. Chaoticity is described as divergence of close-by paths, and entropy is conceptualized as richness of paths emanating from a given present. Where paths end up in the long-term is called the *attractor* of a dynamical system. In systems with fixed-point or periodic attractors, future is rather dull. In chaotic systems however paths are entangled in such a way that bundles which are close-by soon end up far separated and the attractor is called *strange*. Some dynamical systems, and most computational automata, have multiple simple attractors. In these systems while the future is dull, present can be exciting if knowing the now gives little indication on which final simple destiny one ends up in. This is the *basin-centric* way of looking at the dynamics.

**Figure 1:**
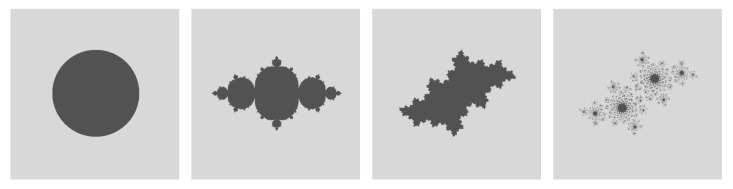
Basin partitioning in (*quadratic Julia sets*) with increasing basin entropy. System parameters $\left(a,b\right)$ from left to right: $\left(0,0\right)$, $\left(-0.75,0\right)$, $\left(-0.35,-0.55\right)$, $\left(-0.40,-0.60\right)$. The last one tends to a disconnected Cantor set as time goes to infinity.

Consider a discrete-time dynamics $x\to x^{2}-y^{2}+a$ and y→2xy+b with (a,b) system parameters. If one marks all possible initial states x(0),y(0) according to two ultimate destiny scenarios (escape to infinity or not), we get the fractal Julia sets as given in Figure 1, and quite interesting ones for different system parameters (a,b). A basin of attraction is the set of initial states that end up in a given attractor. The *basin entropy* measures the entanglement of these basins.

### 2.4. Entropy in Discrete State Space

A cellular automata (‘CA’) is a dynamical system that consists of a grid network of cells where each cell run the same look-up table-based evolution rule that tells it how to change its current state (which is drawn from a finite number of elements) into the next, given the neighbors’ and its current state. The global state of CA is the configuration of local states of each cell on the grid. The local evolution rule and state information exchange between neighbors synchronously update all local states in each time step, changing the configuration of CA to its next, hence generating the dynamics on discrete configuration state space. A CA is called elementary if the grid is 1D, each cell takes binary state values and the neighborhood is just the left-right nearest cells.

Entropy for CA can be defined with the same spirit as Kolmogorov–Sinai entropy given above. Assuming a 1D grid, consider a window of *L* cells and ignore the rest. This is effectively a coarse graining of the configuration space since all configurations that have the same pattern within the window are grouped together. Now, let us look into strings of window patterns generated by the system in *T* time steps $w_{L}^{T}$ with observation frequency $P(w_{L}^{T})$. The entropy of this block (i.e., entropy of the system at this coarse graining) is:

(7) $$H_{L}^{T}=-\sum_{\left\{w_{L}^{T}\right\}}P\left(w_{L}^{T}\right)\log P\left(w_{L}^{T}\right)$$

The equivalent Kolmogorov–Sinai entropy of CA is then given as the average information content per time step of the evolution path with no coarse graining, i.e., when the cell-space window is extended to full grid size:

(8) $$h^{KS}=\lim_{L\to \infty }\frac{1}{L}\lim_{T\to \infty }\frac{1}{T}H_{L}^{T}$$

### 2.5. Dynamics of Cellular Automata

As per Equation (2), Lyapunov exponents rely on differentiability in state space. Since it is a Cantor set, the configuration space of CA does not lend itself to differentiability. Shereshevsky [1] in 1992, proposed the average propagation speed along the CA grid of a single cell disturbance as a proxy of CA’s Lyapunov characterization. Shereshevsky showed that for ergodic 1D CA almost all configurations have unique left and right propagation velocities, $\lambda^{-},\lambda^{+}$, and further established the inequality linking entropy of the CA to entropy of the shift operator σ and the propagation speeds as: $h\left(F\right)\leq h\left(\sigma \right)\left(\lambda^{-}+\lambda^{+}\right)$. Later in 2000, Tisseur [2] expanded the Sheresevsky’s Lyapunov characterization by introducing average propagation velocities.

Entropy given in Equation (8) is finite and calculable for trivial CAs such as left-shifting CA. However, Hurd, Kari and Culik [3] (1992) proved that it is uncomputable for an arbitrary CA. The undecidability result does not preclude the existence of large classes of examples, such as *linear* CA, for which explicit calculations are possible. A CA is called linear if the local state set *S* is endowed with the sum and product operations that make it a commutative ring, and the local evolution function *f* is given as the linear combination of the neighboring cell’s local states, $f\left(x_{-r},\cdots ,x_{+r}\right)=\sum_{i}a_{i}x_{i}$ with $\{a_{i}\}\in S$. With sum and product operations defined on *S*, it is possible to describe a given configuration *c* with characteristic power series $P_{c}\left(z\right)=\sum_{i=-\infty }^{\infty }c_{i}z^{i}$ and the local function as a kernel $A_{f}\left(z\right)=\sum_{i=-r}^{r}a_{i}z^{i}$. For linear CA dynamics reduces to $P_{F(c)}\left(z\right)=P_{c}\left(z\right)A_{f}\left(z\right)$. Manzini et al. published a series of articles [4,5,6,7] on topological dynamics of linear 1D CA and explicitly calculated the entropy.

Topological entropy is a characterization of the richness of time streams of configuration patterns generated by the CA. This richness is closely linked to compressibility of these pattern streams. Excellent progress has been made on practical implementation of compression algorithms for patterns. Lempel–Ziv coding [8] introduced in 1976 is a universal lossless data compression algorithm which is at the core of GIF image format used today. Zenil (2010) [9] proposed to calculate entropy of CA based on compressed length of output patterns with Lempel–Ziv-like compression.

### 2.6. Complexity Classification of Cellular Automata

In the early 1980s, Wolfram ignited research interest in CA with a series of articles [10,11,12] where he classified the apparent spatio-temporal configuration complexity as: (1) tends to a spatially homogeneous state; (2) yields a sequence of simple stable or periodic structures; (3) exhibits chaotic aperiodic behavior; and (4) yields complicated localized structures, some propagating, i.e., *complex patterns*. Wolfram’s classification made a distinction of chaotic vs. complex configuration evolution. This was important because entropy-based definition used in dynamical systems theory did not differentiate chaotic behavior from complex. He conjectured that complex CA performed universal computation. Culik and Yu (1988) [13] proposed a formal framework for Wolfram’s complexity classes. Key demarcation was whether an algorithm existed to decide for any two finite configurations whether one ever evolves to the other. Since then there has been a wide range of classifications on elementary CA to capture the intuition that complex was something different than chaotic.

Li and Packard (1990) [14] looked into the structure of *’rule space’* and investigated bifurcations in the behavior of 1D CA by varying a classifier parameter λ defined as the ratio of non-zero entries in local rule table of CA. The rule space seemed to be split into ordered (fixed or periodic patterns) and chaotic regions with a sharp phase boundary. However, in certain parts of the rule space, between the ordered and chaotic domains, the phase boundary was a thick wall that housed critical rules which showed complex patterns. Kurka (1997) [15] provided three formal ways of classification for 1D CA: (i) based on the complexity of languages generated by partitions of the state space; (ii) based on Lyapunov stability; and (iii) based on attractors of the configuration space. Kurka demonstrated correspondence between the classes of these three schemes and Wolfram’s heuristic classification.

Chua et al. (2002) [16] proposed a complexity index to categorize the rules of elementary CA: Consider a cube graph where each vertex corresponds to one of 2×2×2=8 states of the input triplet of a cell. Let us color each such vertex with Red or Blue depending on what the output state ought to be based on the local rule table. Now, imagine partitioning the cube by planes such that same colored vertices remain in the same partition. Chua’s complexity index is the number of planes required to make such partition. He showed that Class IV elementary CA of Wolfram had index 2 or 3. To overcome certain mis-categorizations of Chua index, Ewert recently (2019) [17] proposed a novel method based on residual circuit complexity. It takes into account not only minimal form representation of the rule table, but also the reduction of input entropy as the system evolves which further reduces the needed minimal form. Applied to elementary CA, Ewert showed that the proposed method matched well to Wolfram’s heuristic classification.

### 2.7. Venturing into General Network Automata

Very few attempted to go beyond classical cellular automata which has two built-in regularities: (i) cells are arranged on a regular grid-like cell space, and (ii) each cell executes the same local rule, i.e., homogeneous. These properties arguably give the classical CA a physical-like feel with uniform laws and regular space, and make dynamical analysis tractable. It is, however, tempting to go beyond classical set up and consider generalized graph automata where cells are placed at vertices of a general directional graph, and each executes a different local rule. Many real-life systems with interacting agents are typically organized into networks such as Watts–Strogatz type small-world graphs, or Barabasi–Albert type scale-free graphs with non-uniform local rules.

Marr and Hutt (2005) [18] investigated the pattern formation capacity of binary graph automata where each cell was executing a local law based on the average state value of the neighbors passed through a threshold function parametrized by value κ. They investigated both small-world and scale-free type graphs, varying the graph incrementally to see impact on pattern formation. They measured Shannon entropy of time strings of a single cell averaged over all cells. Since graphs were not regular grids, the authors did not define any spatial pattern concept, and investigated time patterns of single cells only.

Tomassini (2006) [19] analyzed the performance of binary graph automata with homogeneous cell rules over small-world and scale-free graphs over two tasks. In density classification task, the cells, starting from random configurations are expected to converge to all 1 if their initial 1 density were greater than 0.5 and vice-versa. In synchronization task, the cells starting from random initial configurations are expected to synchronize into a global flip-flop cycle. Cattaneo et al. (2009) [20] introduced two types of non-homogeneous 1D CA: (i) local rules are variable only in a region outside which the rule is uniform, and (ii) the rule is non-uniform everywhere but the neighborhood has always same radius. The authors showed that most topological properties get demolished when CA becomes non-homogeneous.

### 2.8. Back to Basins

Basin entropy defined in Section 2.3 is a relatively new concept and plays a central role in this paper. Daza et al. (2016) [21] calculated basin entropy of two well-known physical dynamical systems with path chaoticity: (i) Duffing oscillator which is a periodically driven rod with a nonlinear elasticity, and (ii) Hénon-Heiles system which describes motion of a star around a galactic center. They defined basin entropy by coarse graining the basin into boxes and marking distribution of ultimate destinies’ probability in each box, to which they applied an entropy measure.

Finite-size discrete systems have been mostly dismissed in the entropy world since paths are of finite size and ultimately fixed or periodic. However, these systems as computational machines are very rich in the structure of their basins of attraction. In fact, mapping initial states to ultimate destinies is what computation means for these machines. As we shall now formally see, our definition of chaoticity and more importantly complexity of such finite-state machines is all about quantifying the structure of basin partitioning, and as such fundamentally differs from previous work on complexity and entropy for automata.

## 3. Computron

*“Things do not exist other than in arrangements”*. Tractatus—Ludwig Wittgenstein

### 3.1. Construction

A computron $M=[G,C,v_{\mathrm{IN}},v_{\mathrm{OUT}}]$ is an arrangement of *N* cells with binary states $\omega_{i}$ which is formally constructed with:

1. A *generating graph* G(V,E) where each cell is assigned to a vertex $v_{i}\in V$. A cell $v_{j}$ is said to be a neighbor of cell $v_{i}$ if there exists the directional edge $e_{ji}\in E$. The ordered set of neighbor cells of $v_{i}$ is called its neighborhood denoted as $\delta_{i}=\left[v_{a},v_{b},\cdots \right]$. Each cell is assumed to be also its own neighbor, i.e., $e_{ii}\in E,\forall i$.
2. A set of *connectors* $C=\{c_{i}\}$, each assigned to a vertex. A connector $c_{i}\left(\Omega_{i}\right)$ is a binary valued look-up table based on the state configuration of neighbors, $\Omega_{i}=\left[\omega_{j}|v_{j}\in \delta_{i}\right]$.
3. An *input cell* at vertex $v_{\mathrm{IN}}$ which acts as interface for the external binary input σ, if the computron is driven with an external signal, $\omega_{\mathrm{IN}}=\sigma$.
4. A nominated *output cell* at vertex $v_{\mathrm{OUT}}$ which provides a binary output γ if required, $\gamma =\omega_{\mathrm{OUT}}$.

**Figure 2:**
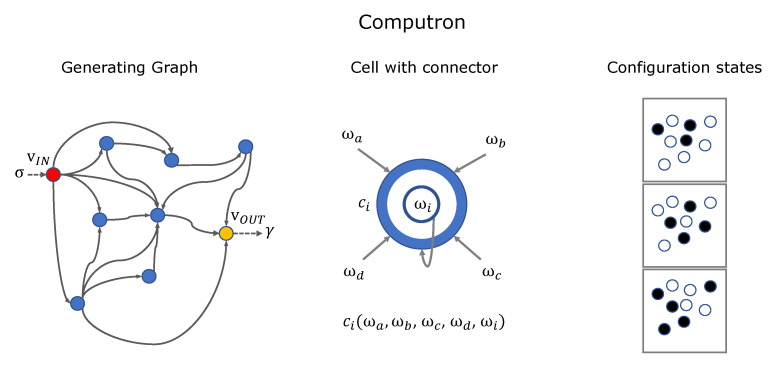
Computron is constructed on a generating graph. Cells are placed at vertices of the graph. Each cell has a binary state $\omega_{i}$ and is endowed with a local look-up table rule, called a connector. Global state of the computron is the configuration of local states on the generating graph. $v_{\mathrm{IN}}$ is the special cell that interfaces the external input and $v_{\mathrm{OUT}}$ is the nominated cell for reading the output.

Figure 2 depicts the construction of a computron. The local states are updated synchronously. The global state of computron $s=[\omega_{0},\omega_{1},\cdots ,\omega_{N-1}]$ is the configuration of local states on the generating graph. Local dynamics induce the global dynamics of the computron as follows:

(9) $$\begin{array}{cc} \omega_{i}^{n} & \to \omega_{i}^{n+1}=c_{i}\left(\Omega_{i}^{n}\right)\\ s^{n} & \to s^{n+1}=\left[\omega_{0}^{n+1},\omega_{1}^{n+1},\cdots ,\omega_{N-1}^{n+1}\right]=:T\left(s^{n}\right) \end{array}$$

An autonomous computron performs computation by mapping an initial configuration to an attractor (fixed or periodic). Such mapping partitions the configuration space. A driven computron generates an output string for a given input string based on an initial starting configuration.

### 3.2. Computron vs. CA and FSA

By construction, any finite-size cellular automata is a computron where generating graph is a regular grid, connectors are homogeneous, and there is no external input. Finite-state automata (‘FSA’) is a computational machine that maps an input string to an output one with no memory. Formally, the machine $M=[S,s_{0},\Sigma ,\Gamma ,T,U]$ is specified by a set of states *S*, an initial state $s_{0}$, an input alphabet Σ, an output alphabet Γ, the *Transition Map* T:S×Σ→S, and the *Output Map* U:S×Σ→Γ. FSA generates an output string $y=\gamma_{0}\gamma_{1}\gamma_{2}...\in \Gamma^{*}$ driven by the input string $x=\sigma_{0}\sigma_{1}\sigma_{2}...\in \Sigma^{*}$. Without loss of generality we assume that input and output alphabets are binary.

**Lemma** **1.** *For any FSA with $2^{N}$ states there is an equivalent computron of N cells plus the input cell.*

**Figure 3:**
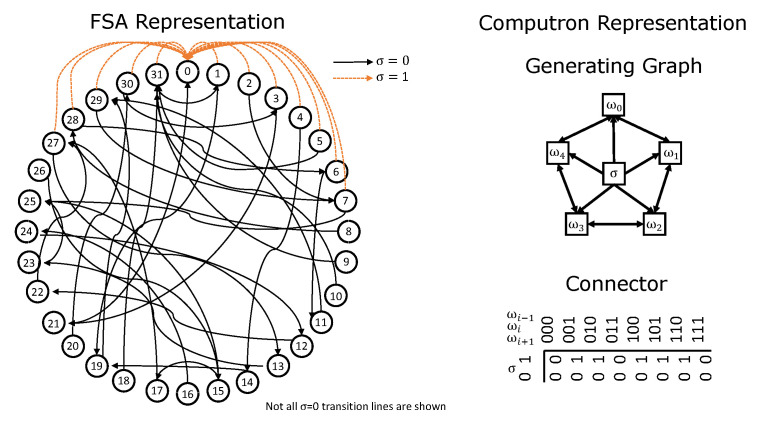
32-state machine: Wolfram cellular automata Rule 110 on 5-circle and its equivalent computron with external input used as Reset (0) or Step (1)

Figure 3 shows the state transition diagram of 32-state machine and its equivalent computron representation with 5 cells. We note that state transition diagram of finite-state automata lacks intuitive description of the inherent structure provided by the equivalent computron. The generating graph of the computron defines *‘local flow of information’* whereas connectors of each unit describe *‘local processing of information’*.

## 4. Diversity Space

### 4.1. Purpose

Dynamical systems live on metric spaces whereas computation does not. Computron as a model of computation is constructed with global states defined as configurations of local states on its generating graph. In this section, we formally define a set measure called *diversity measure* which gives the cardinality of a set adjusted for similarity of its elements. Diversity measure induces a similarity distance between two sets as the measure of their symmetric difference. This similarity metric generates a metric space for the states of the computron called *diversity space*. We later perform all our dynamics investigations for the computron on this diversity space.

Various distance functions between sets with elements embedded in a metric space have been proposed in the literature. *Hamming* distance is arguably the simplest one which counts the number of different elements in two sets; it lacks the concept of similarity among the elements. *Hausdorff* distance between two sets is calculated as the furthest separation of one set’s elements from closest of the other; final result reduces to distance between two most significant elements disregarding the rest.

(10) $$d_{h}\left(S_{1},S_{2}\right)=\max\left\{\max_{e\in S_{1}}\min_{f\in S_{2}}\Delta \left(e,f\right),\max_{e\in S_{2}}\min_{f\in S_{1}}\Delta \left(e,f\right)\right\}$$

The *sum-of-min-distances* incorporates all elements, but it is an aggregation of closest peers, hence presence of non-closest peers are shadowed, thus fails to capture an ensemble closeness.

(11) $$d_{m}\left(S_{1},S_{2}\right)=\frac{1}{2}\left(\sum_{e\in S_{1}}\Delta_{m}\left(e,S_{2}\right)+\sum_{e\in S_{2}}\Delta_{m}\left(e,S_{1}\right)\right)\;$$

Other distance measure between sets have been proposed to capture the overall similarity of the elements of two sets given the underlying metric space such as *Surjection* distance and *Link* distance [22]. Bennett, Vitanyi and Zurek et al. [23] introduced *absolute information distance* metric between two objects. Consistent with definition of absolute information content of an object as length of the shortest binary program K(x) running on a universal machine, generating the given object *x*, they described the distance metric between two given objects *x* and *y* as length of the shortest binary program that generates one given the other: $d^{*}\left(x,y\right)=\max K\left(x|y\right)+\max K\left(y|x\right)$. They proved that absolute information distance is a universal pattern similarity metric. This theoretical metric is similar to that of algorithmic complexity in that while universal, it is not computable in its pure form.

### 4.2. Diversity Measure

We introduce *diversity measure* as the size of a set adjusted for the similarity of its elements. The intuition behind set-theoretic formulation below is as follows: Assume we are asked to form a *diverse team* from a pool of students. Each student has a profile which allows us to define how similar two students are. We build the team by adding one student at a time. First member contributes an *individuality* of 1. Second member’s individuality is reduced by her similarity to the first member. Third member’s is reduced by his similarity to the first two. Diversity of the team is sum of individualities.

Let (Q,f) be a metric space where *Q* is a finite set and *f* is a metric of *Q*, i.e., a function $f:Q\times Q\to R^{0+}$ such that for any x,y,z∈Q, (i) f(x,y)=0⇔x=y, (ii) f(x,y)=f(y,x), and (iii) f(x,z)≤f(x,y)+f(y,z). Let $\rho :R^{0+}\to \left[0,1\right]$ be a concave monotonic function with ρ(0)=0 (*similarity kernel*) and define ρ(x,y)=ρ(f(x,y)), then (Q,ρ) is also a metric space.

*Individuality* of an element *x* given a set of constraining elements *A* is defined as:

(12) $$d\left(x|A\right)=\prod_{y\in A}\rho \left(x,y\right)$$

if ρ(x,y)=0, i.e., there exists an element y∈A that is exactly the same as *x*, then d(x|A)=0, meaning *x* has no individuality given the constraining set *A*.

*Diversity* of an ordered set $\vec{A}=\left[x_{0},x_{1},x_{2}\cdots \right]$ given a set of constraining elements, *B* is defined as:

(13) $$\begin{array}{cc} d(\vec{A}|B) & =d(x_{0}|B)\\ \; & +d(x_{1}|\left\{x_{0}\right\}\cup B)\\ \; & +d(x_{2}|\left\{x_{0},x_{1}\right\}\cup B)\cdots \end{array}$$

Clearly, the ordering of the tuple $\vec{A}$ matters for the calculation of diversity, therefore we define the *diversity measure* of a set *A* constrained by set *B* as the supremum over all possible orderings of the tuple $\vec{A}$. For numerical experiments, we devised a routine whose computation load grows quadratically rather than combinatorially which made calculation of diversity measure feasible.

(14) $$∥A|B∥=\underset{\vec{A}}{\sup}d\left(\vec{A}|B\right)$$

(15) $$\begin{array}{cc} d(\vec{A}) & =d(\vec{A}|\emptyset )\\ ∥A∥ & =∥A|\emptyset ∥ \end{array}$$

Section 6 of this paper introduces probabilistic computron which generates probability distributions on states. Therefore, we extended diversity measure to handle *weighted sets*. Let $w:S\to R^{0+}$ be a weight function. *Individuality* of a weighted element *x* given a set of constraining weighted elements *A* is given as:

(16) $$d_{w}\left(x|A\right)=w\left(x\right)\prod_{y\in A\backslash x}\rho \left(x,y\right)$$

Diversity and diversity measure for weighted sets follow the same definitions as in Equations (13) and (14) using individuality for weighted sets. We use the following extension for union $\tilde{\cup }$, symmetric difference $\tilde{▵}$, and subset $\tilde{\subseteq }$:

(17) $$\begin{array}{cc} A\tilde{\cup }B & =\{x:x\in A\cup B,w_{C}\left(x\right)=\left(w_{A}\left(x\right)+w_{B}\left(x\right)\right)\}\\ A\tilde{▵}B & =\{x:x\in A\cup B,w_{C}\left(x\right)=|w_{A}\left(x\right)-w_{B}\left(x\right)|\}\\ A\tilde{\subseteq }B & \Leftrightarrow (x\in A\Rightarrow x\in B,w_{A}\left(x\right)\leq w_{B}\left(x\right)) \end{array}$$

**Lemma** **2** (Diversity measure)**.** *Diversity is a sub-additive measure on Q.*

(18) $$A\tilde{\subseteq }B\Rightarrow {∥A∥}_{w}\leq {∥B∥}_{w}$$

(19) $$∥A_{0}\tilde{\cup }A_{1}{∥}_{w}\leq ∥A_{0}{∥}_{w}+{∥A_{1}∥}_{w}$$

*Diversity-induced distance* (DiD) is an induced metric on $Q^{*}$ that captures dissimilarity between two sets, $A,B\in Q^{*}$:

(20) $$\varphi \left(A,B\right)=\frac{∥A\tilde{▵}B∥}{\left|Q\right|}$$

where $\varphi :Q^{*}\times Q^{*}\to \left[0,1\right]$, $\tilde{▵}$ is the symmetric difference operator as defined in Equation (17) and $\left|Q\right|=\sum_{Q}w\left(x\right)$ is size of weighted space.

**Lemma** **3** (DiD metric)**.** $\left(Q^{*},\varphi \right)$ is a metric space.

### 4.3. Entanglement

We introduce the entanglement concept which will later be used in defining partitioning complexity of a computron. *Entanglement* of a set of sets $A=\{A_{0},A_{1},A_{2}\cdots \}$ is defined as:

(21) $$∥A∥=\underset{\vec{A}}{\sup}D\left(\vec{A}\right)$$

where

(22) $$D\left(\vec{A}\right)=∥A_{0}∥+∥A_{1}|A_{0}∥+∥A_{2}|A_{1},A_{0}∥\cdots$$

**Figure 4:**
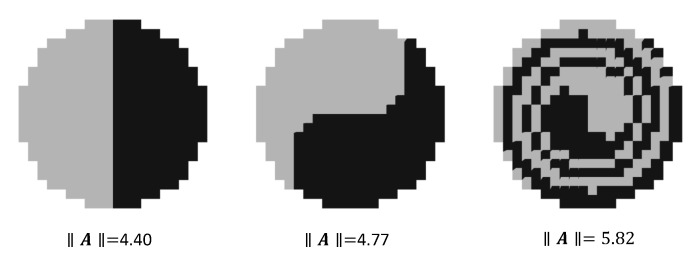
Illustration of entanglement: Each element is assumed to be a pixel with Manhattan distance metric. Space is partitioned into two sets, $A=\{A_{0},A_{1}\}$. Three different partitions with increasing entanglement measure is provided.

Entanglement captures the total diversity of a collection of sets where each set’s diversity is constrained by the presence of the other sets. The more the sets are within each other’s proximity entanglement increases. On the other hand, as one given set’s elements get separated from each other, that set’s diversity decreases. Entanglement captures the balancing of these two factors in calculating the total diversity of the set of sets. This is illustrated in Figure 4.

**Lemma** **4** (Maximal entanglement)**.** *Let $P=\{B_{0},B_{1}...\}$ be a partitioning of Q (i.e., $\cup_{i}B_{i}=Q$, and $B_{i}\cap B_{j}=\emptyset ,i\neq j$) with $P^{0}=\left\{Q\right\}$ (nil partition) and $P^{\infty }=\left\{\left\{x_{0}\right\},\left\{x_{1}\right\},\cdots \right\},\forall x_{i}\in Q$ (full partition), then:*

(23) $$∥P^{0}∥=∥P^{\infty }∥\geq ∥P∥$$

## 5. Dynamics of Autonomous Computrons

### 5.1. Embedding in Metric Space

Given a computron M=[G,C] with a generating graph *G* and connectors set *C*, we embed it in a metric space that captures similarity of its configuration states as follows: The distance between two cells is set as the *shortest-path-length* on generating graph between the two vertices occupied by these cells mapped to a unit interval. The intuition of this distance definition between two cells is that a state change in one of the cells can possibly impact the state of other one in compute steps that are no less than the shortest-path-length.

(24) $$d\left(v_{i},v_{j}\right):=\rho \left(\mathrm{SPL}\left(v_{i},v_{j}\right)\right)$$

$\rho :R^{0+}\to \left[0,1\right]$ concave, monotonic with ρ(0)=0. We then define the configuration state of the computron as set of cells that have local state of 1.

(25) $$s\in S:=\{v_{i}|\omega_{i}=1\}$$

This leads to diversity-induced distance as a metric for dissimilarity of the states for computron:

(26) $$\varphi \left(s_{0},s_{1}\right):=\frac{∥s_{0}▵s_{1}∥}{\left|S\right|}$$

**Figure 5:**
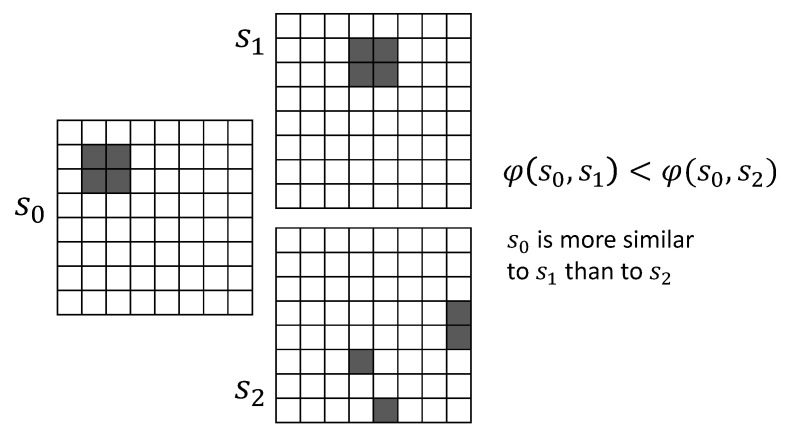
$s_{0},s_{1},s_{2}$ are configurations of a computron with 2D generating graph (binary images). Diversity-induced distance uses diversity measure to define similarity between configurations.

By Lemma 3, (S,φ) is a metric space with each configuration of the computron an element of the space. This is the *diversity space* that we embed the computron where distance between two global states of the machine is based on the similarity of the corresponding configurations. Figure 5 illustrates the concept on a computron where generating graph is assumed to be regular 2D grid. Each configuration is a set of pixels that are have state 1 (a binary image). Diversity-induced distance between configuration $s_{0}$ and $s_{1}$ is less than that of $s_{0}$ and $s_{2}$, capturing their similarity, although Hamming distances are the same.

### 5.2. Computron as a Dynamical System

An autonomous computron has no external input. Starting from an initial configuration, it moves to next based on the transition map given in Equation (9). The dynamics is governed by discrete-time iteration on configuration metric space (S,φ):

(27) $$\begin{array}{cc} s^{n+1} & =T(s^{n})\\ T^{n+1}: & =T\circ T^{n},T^{0}=I,n\in N \end{array}$$

An *attractor* of the computron is a tuple of configurations $\vec{A}=\left[s_{0},s_{1},..s_{p-1}\right]$ where $s_{i+1}=T\left(s_{i}\right)$, $s_{0}=T\left(s_{p-1}\right)$ and $s_{n}\neq s_{m}\Leftrightarrow n\neq m$. For p=1, it is called a fixed-point attractor with the corresponding state a *‘fixed-state’*, else a periodic attractor. States that are elements of a periodic attractor are called *periodic states*. States that are not element of an attractor are called *transient states*. The *basin* of an attractor are those states that iterate into, or a part of, that attractor $B\left(\vec{A}\right)=\left\{s\in S:\exists k∋T^{k}\left(s\right)\in \vec{A}\right\}$.

**Lemma** **5** (Basin partition)**.** *The set of all basins of an autonomous computron M is a partition of its configuration space S, denoted as $P_{M}=\left\{B_{0},B_{1},\cdots \right\}$, with the corresponding attractors $A_{M}=\left\{\vec{A_{0}},\vec{A_{1}},\cdots \right\}$ where $B_{i}\subset S$, $\cup_{i}B_{i}=S$ and $B_{i}\cap B_{j}=\emptyset ,i\neq j$.*

**Figure 6:**
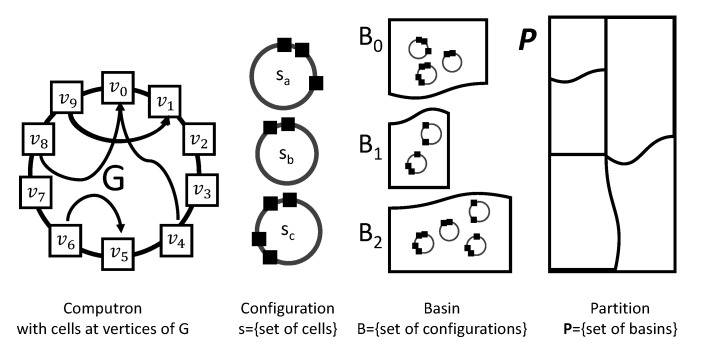
Dynamics of a computron is characterized by the structure of its basins of attraction. A partition is a set of basins; a basin is a set of configurations; a configuration is a set of cells that have local state 1.

Figure 6 gives a recap: A computron consists of a set of cells placed at vertices of a graph *G* with binary local states $\left\{\omega_{i}\right\}$. The configuration of the machine is specified as set of cells that are have local state 1. A basin is a set of configurations. As per Lemma 5, the configuration space is partitioned into basins by the computation performed by the computron.

### 5.3. Chaotic Computation

As defined in Equation (2) Lyapunov exponents characterize the rate of separation (or convergence) of infinitesimally close trajectories in a dynamical system. In discrete-time systems (maps), $x_{n+1}=f\left(x_{n}\right),n\in N$, for an orbit starting with initial point $x_{0}$, Lyapunov exponent becomes:

(28) $$\lambda \left(x_{0}\right)=\lim_{n\to \infty }\frac{1}{n}\sum_{i=0}^{n-1}\ln\left|f^{\prime }\left(x_{i}\right)\right|$$

For a discrete-time discrete state-space dynamical system (i.e., dynamics of autonomous computron) on a metric space (S,φ), we define Lyapunov number for an initial configuration $s_{0}\in S$ as follows: Let $B_{\epsilon }\left(s\right)=\left\{q\in S:\varphi \left(s,q\right)<\epsilon \right\}$ denote the epsilon-ball around state *s*, where ϵ is set as the lowest value where epsilon-ball is non-empty. Let $A_{M}=\left\{A_{0},A_{1},\cdots \right\}$ be the attractors of computron, and $P_{M}=\left\{B_{0},B_{1},\cdots \right\}$ the corresponding basins of attraction. We calculate Lyapunov number of a state as the average separation of its neighboring states as iteration time goes to infinity. Since, each state converges to its attractor, we measure the dissimilarity of attractors using an induced DiD metric.

(29) $$\lambda \left(s\right)=\frac{1}{|B_{\epsilon }\left(s\right)|}\sum_{q\in B_{\epsilon }\left(s\right)}\frac{\Phi (A_{k},A_{l})}{\Phi (\{s\},\{q\})}$$

$$s\in B\left(A_{k}\right),q\in B\left(A_{l}\right)$$

Lyapunov number for the computron is the average over the configuration space $\lambda_{M}:={\langle \lambda \left(s\right)\rangle }_{S}$.

### 5.4. Complex Computation

While chaos is a well-defined concept in dynamical systems theory, complexity has been more elusive with wide range of definitions as reviewed by Grassberger [24]. Observer’s reference base using existing information is relevant for defining complexity, yet it leads to observer subjectivity. This can be avoided by either dealing with an equivalence class of the object as the reference base, or by treating the object on a self-referential basis using a generative description. In the former case, we are in the domain of information theoretical definition of complexity, whereas the latter is in the domain of algorithmic complexity. Either way, the complexity becomes a measure of difficulty in describing the object in question with regards to a reference base.

**Figure 7:**
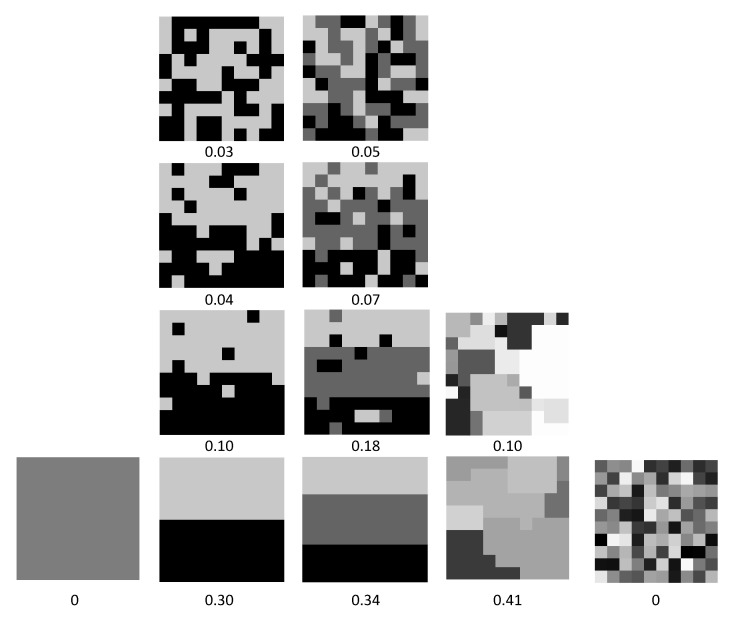
Illustration of diversity-based complexity measure for various partitionings. Nil partitioning and infinite partitioning has zero complexity. Complexity first increases with increasing number of partitions then starts to decrease. For a given number of partitions increasing entanglement reduces complexity. Complexity is maximized somewhere between order and chaos.

We introduce *diversity-based complexity* of a computron as the disentanglement of its basin partition. This is a basin-centric approach, not a path-centric one. Computron performs its computation by act of mapping the input configurations to attractors, generating a partitioning of configurations space into basins of attraction. We consider the complexity of this partitioning as the defining characteristic of computron as the computational machine. This is different than Wolfram’s complexity definition which is path-centric.

(30) $$\Psi_{M}=1-\frac{∥P_{M}∥}{∥{P^{\infty }}_{M}∥}$$

with $\Psi_{M}\in \left[0,1\right]$ since $∥P_{M}∥\leq ∥{P^{\infty }}_{M}∥$ by Lemma 4. This definition means that a complex computron has a basin partition where each basin consists of maximally diverse set of configurations with respect to other configurations in that basin, and furthermore basins themselves are maximally diverse with respect to each other. Therefore, a high complexity partitioning of the configuration space requires maximum information to describe such partitioning. As depicted in Figure 7 complexity of a partitioning increases initially with the number of partitions, then it starts decrease such that no partitioning and full partitioning have zero complexity.

**Lemma** **6** (Minimal complexity)**.** *Absorbing and idle computrons have the least complexity (nil) compared to any other computron.*

(31) $$\Psi_{M^{0}}=\Psi_{M^{\infty }}=0\leq \Psi_{M}$$

An *absorbing* computron has a single fixed-point attractor which all configurations iterate into denoted as $M^{0}:\forall s\in S,\exists k\in N∋T^{k}\left(s\right)=s^{*}$, where *T* is the transfer map of computron and $s^{*}$ is the absorbing state. An *idle* computron does nothing, i.e., each state is mapped to itself; and is denoted as $M^{\infty }:\forall s\in S,T\left(s\right)=s$.

### 5.5. Case Study—1D Cellular Automata

**Figure 8:**
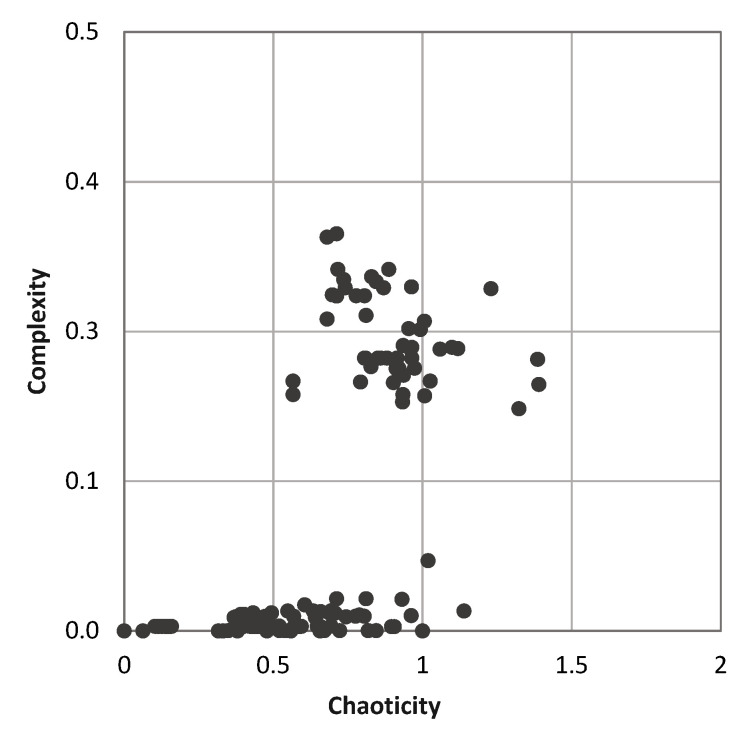
Chaoticity λ vs complexity Ψ of the universe of 32 state computrons with linear cyclic generating graph and homogeneous connectors

We analyzed complexity and chaoticity of autonomous computrons with linear cyclic generating graph and homogeneous connectors, i.e., elementary cellular automata. As expected, absorbing and idle computrons have minimal complexity and chaoticity. With same generating graph (linear cyclic of 5 cells), we analyzed different connector types. Chaoticity and complexity of each computron is plotted in Figure 8. There are several elementary CA that have very low complexity and not chaotic (less than 1 chaoticity). However, there is a group of machines that demonstrate high complexity while not chaotic.

## 6. Dynamics of Driven Computrons

### 6.1. Computron as a Markov Chain

A *driven computron*, unlike an autonomous one, has a transition map that is a function of its current configuration and the external input drawn from binary alphabet. Dynamics is governed by discrete-time iteration:

(32) $$s^{n+1}=T\left(s^{n},\sigma^{n}\right)=\left\{\begin{array}{cc} T^{A}\left(s^{n}\right) & \sigma^{n}=0\\ T^{B}\left(s^{n}\right) & \sigma^{n}=1 \end{array}\right.$$

where $\sigma^{n}$ is the external input at time *n*. We assume no a priori information about the input string other than observed frequency of alphabet letters, resulting in representing the input string as a sequence of independent identically distributed random variables on binary alphabet Σ with p=Pr{σ=0}.

A *noisy computron* is a computron exposed to thermal fluctuations in the binary states of its cells. The dynamics is governed by probabilistic discrete-time iteration:

(33) $$\begin{array}{cc} s^{n+1} & =T^{\varepsilon }\left(s^{n},\sigma^{n}\right)\\ \; & =\left\{\begin{array}{cc} T^{A}\left(s^{n}\right) & \mathrm{with}\;\mathrm{prob}.\;p(1-\varepsilon )\\ T^{B}\left(s^{n}\right) & \mathrm{with}\;\mathrm{prob}.\;(1-p)(1-\varepsilon )\\ s_{r}\in B_{\epsilon }\left(s^{n}\right) & \mathrm{with}\;\mathrm{prob}.\;\varepsilon \end{array}\right. \end{array}$$

where $B_{\epsilon }\left(s^{n}\right)$ is epsilon-ball neighborhood of configuration $s^{n}$ and ϵ is the probability of malfunction due to thermal noise.

We now enter the domain of Markov chains to describe the probabilistic dynamics of the driven and noisy computrons. A *discrete Markov chain* is a sequence of random variables with domain *S*, $\left\{X_{n},n\in N\right\}$, where each random variable $X_{n}$ is independent of prior random variables in the sequence given its previous one.

(34) $$Pr\left\{X_{n}=j\;|\;X_{n-1}=i,X_{n-2}=k,\cdots \right\}=P_{ij}$$

Probabilistic computrons are equivalent to a discrete Markov chain. They can be represented with directed labeled graphs where each vertex is assigned a configuration $s_{i}\in S$, and an edge from configuration $s_{i}$ to $s_{j}$ with transition probability $P_{ij}\neq 0$. It can also be represented by a M×M*transition probability matrix*P where M=|S| is the number of all configurations and $P_{ij}=P_{ij}$. From matrix representation, it follows that probability of reaching *k* from *i* in exactly *n* steps is $P_{ik}^{n}$.

#### Terminology

- n-step *walk* is an ordered set of states $\left(i_{0},i_{1},\ldots ,i_{n}\right)$ in which there is a directed edge from $i_{m-1}$ to $i_{m}$ and no states are repeated
- State *j* is *accessible* from state *i* if there exists a walk from *i* to *j*, shown as i→j
- Two states *communicate* if i→j and j→i
- *Class* is a set of states where each pair of states in the set communicate, and none communicate with a state outside the class
- State *i* is called *recurrent* if i→j⇒j→i
- *Transient* state is a state that is not recurrent
- *Period* of a state *i* is the greatest-common-divisor of those values of *n* for which $P_{ii}^{n}>0$. If the period is 1, the state is called *aperiodic*.
- It follows from definitions that for a given class, either all states are recurrent, or they are all transient. It can also be shown that all states in the same class have the same period.
- A class is called *ergodic* if it is recurrent and aperiodic.
- A Markov chain consisting entirely of single ergodic class is called and *ergodic chain*.
- A *unichain* contains a single recurrent class plus, possibly, some transient states. An *ergodic unichain* is a unichain for which the recurrent class is ergodic.
- A *steady-state distribution* for an *M* state Markov chain with transition matrix P is a row vector π that satisfies:
  (35) $$\pi =\pi P,\;\mathrm{where}\;\pi_{i}\geq 0,\Sigma_{i}\pi_{i}=1$$

For a given Markov chain, Equation (35) always has a solution, and the solution is unique if and only if the Markov chain is a unichain. If there are *c* recurrent classes, then there are *c* linearly independent solutions each with non-zero entries only for the states of corresponding recurrent class. $P^{n}$ converges if and only if recurrent classes are aperiodic with each row of $P^{n}$ converging to π of one class.

We compare dynamical systems concepts for autonomous computron to that of Markov chain. An attractor is analogous to a recurrent class. An ergodic dynamical system has a single attractor, and similarly an ergodic chain has a single recurrent aperiodic class. In autonomous computron a transient state feeds into a single attractor, therefore the concept of basin of attraction is well-defined. However, in driven automaton, a transient state can feed into multiple recurrent classes with overlapping basins.

Let $P_{ij}$ be the probability of transition from state $s_{i}$ to $s_{j}$ as described in Equation (33), and P the corresponding transition probability matrix. Let $p_{n}\left(s_{i}\right)$ be the probability of computron being in configuration $s_{i}$ at time *n*, and $P_{n}$ be the corresponding probability distribution vector over configuration space *S*. Its evolution starting from an initial distribution $P_{0}$ is governed by the *master equation*:

(36) $$p_{n+1}=P_{n}P$$

*Impact set*$R_{s}$ of a given configuration of computron is a set weighted by the impulse response distribution $\pi_{s}$ defined as follows:

(37) $$\begin{array}{cc} \pi_{s} & =\lim_{n\to \infty }p_{s}^{\delta }P^{n}\\ p_{s}^{\delta }\left(x\right) & =\left\{\begin{array}{cc} 1 & \mathrm{if}\;x=s\\ 0 & \mathrm{else} \end{array}\right. \end{array}$$

(38) $$R_{s}=\left\{x\in S:\pi_{s}\left(x\right)>0\right\}$$

Since Equation (36) is linear, steady-state distribution of a driven computron can be written as linear combination of the impulse responses of all states weighted by the initial probability distribution. Let $P_{0}$ be an arbitrary initial distribution stated as linear combination of delta–dirac distributions over all configurations:

(39) $$p_{0}=\sum_{s\in S}p_{0}\left(s\right)p_{s}^{\delta }$$

Then, steady-state distribution π becomes:

(40) $$\pi =\sum_{s\in S}p_{0}\left(s\right)\pi_{s}$$

### 6.2. Ergodic Decomposition

A computron is said to be ergodic if its Markov chain representation is ergodic (i.e., single recurrent aperiodic class). Typically, computrons are non-ergodic, i.e., they have more than one recurrent class, and can be periodic. A periodic computron would not have a stationary steady-state distribution as $P^{n}$ would not converge. Stationarity can be introduced by imposing *‘asynchronicity’* where we modify each state’s transition map with non-zero probability λ to *stay idle*.

(41) $$\tilde{P}=\left(1-\lambda \right)P+\lambda I$$

As per the definition of periodicity (see Terminology), the greatest-common-divisor of *n* for which ${\tilde{P}}_{ii}^{n}$ becomes 1 for all states (since they all have a transition to self with λ probability), and therefore the machine would become aperiodic. An aperiodic computron would have ${\tilde{P}}^{n}$ converge with each row becoming $\tilde{\pi }$ of one recurrent class.

The Coloring Lemma provides a technique for identifying the recurrent classes $C_{M}=\left\{C_{1},C_{2},\cdots ,C_{K}\right\}$ of a computron. Let $P_{a},P_{b}$ two random initial distributions with non-zero entries for each state, and let $\pi_{a}$ and $\pi_{b}$ be the corresponding steady-state distributions in an asynchronous computron. Define, the *‘gray color’* of a state c(s) as:

(42) $$c\left(s\right)=\left\{\begin{array}{cc} \pi_{b}\left(s\right)/\pi_{a}\left(s\right) & \mathrm{if}\;\pi_{a}\left(s\right)>0\\ 0 & \mathrm{else} \end{array}\right.$$

**Lemma** **7** (Coloring)**.** (*Two states will, almost always, have the same shade of gray if and only if they are elements of same recurrent class, provided all classes of the computron are ‘distinguishable’ (as defined in the proof); i.e., for two random non-zero initial distributions, $c\left(s\right)=c\left(q\right)>0\Leftrightarrow s,q\in C_{i}$, almost always.*

Having identified the recurrent classes of a computron using the Coloring Lemma, we now use them as basis for ergodic decomposition:

**Lemma** **8** (Ergodic bases)**.** *Recurrent classes form a non-overlapping spanning set (basis) for the impact sets of all states. Let $C_{M}=\left\{C_{1},C_{2},\cdots ,C_{K}\right\}$ be the set of recurrent classes of a computron and $\chi_{s}=\left\{\chi_{1}\left(s\right),\chi_{2}\left(s\right),\cdots \chi_{K}\left(s\right)\right\}$ the ergodic decomposition of impulse response $\pi_{s}$:*

(43) $$\chi_{k}\left(s\right)=\sum_{q\in C_{k}}\pi_{s}\left(q\right)$$

*then:*

(44) $$\sum_{k}\chi_{k}\left(s\right)=1,\;\mathrm{with}\;\chi_{k}\left(s\right)\geq 0,\forall s\in S$$

We can now formally define *basin of attraction for a recurrent class* as a weighted-set $\tilde{B}\left(C_{k}\right)$:

(45) $$\tilde{B}\left(C_{k}\right)=\left\{s:\chi_{k}\left(s\right)>0,w_{\tilde{B}}\left(s\right)=\chi_{k}\left(s\right)\right\}$$

**Lemma** **9** (Fuzzy partition)**.** *The set of basins of attraction of a probabilistic computron is a fuzzy partition of its configuration space, denoted as ${\tilde{P}}_{M}=\left\{\tilde{B}\left(C_{1}\right),\tilde{B}\left(C_{2}\right),\cdots ,\tilde{B}\left(C_{K}\right)\right\}$. A fuzzy partition of S is the set of weighted sets $\tilde{P}=\left\{{\tilde{B}}_{1},{\tilde{B}}_{2},..{\tilde{B}}_{K}\right\}$, where $\cup_{i}{\tilde{B}}_{i}=S$, and $\sum_{k}w_{{\tilde{B}}_{k}}\left(s\right)=1,\forall s\in S$.*

### 6.3. Chaoticity—Complexity of Probabilistic Automata

A computron acting on a configuration space transforms each configuration into its impact set. Configurations that are similar to each other (based on diversity-induced distance) can be transformed to similar or different impact sets. We define the ratio of DiD distance among the impact sets of two configuration to distance between them as the *‘bending ratio’*. Computron’s action on the configuration space can be conceptualized as transforming the distance between each pairs of configurations with the bending ratio.

(46) $$\tau \left(s,q\right)=\frac{\tilde{\Phi }\left(R_{s},R_{q}\right)}{\Phi (\{s\},\{q\})}$$

Lyapunov number for a state is the average bending of the ϵ-ball around such state:

(47) $$\lambda \left(s\right)=\frac{1}{|B_{\epsilon }\left(s\right)|}\sum_{q\in B_{\epsilon }\left(s\right)}\tau \left(s,q\right)$$

Chaoticity of the machine is measured by the average Lyapunov number over configuration space. $\lambda_{M}:={\langle \lambda \left(s\right)\rangle }_{S}$.

As an extension of the complexity definition we used for autonomous computrons, Equation (30), complexity of a probabilistic computron is the disentanglement of its fuzzy basin partition.

(48) $${\tilde{\Psi }}_{M}=1-\frac{∥{\tilde{P}}_{M}∥}{∥{\tilde{P}}_{M}∥}\in \left[0,1\right]$$

**Figure 9:**
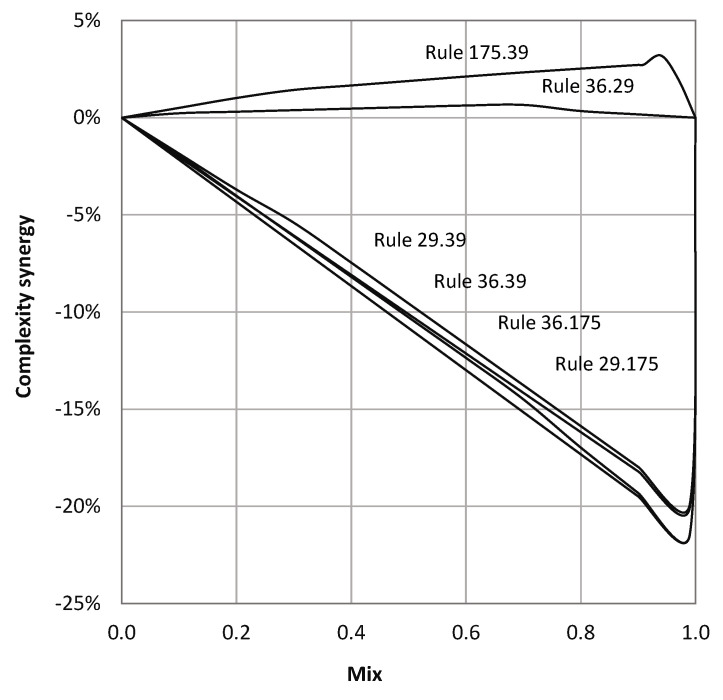
Increase (decrease) in complexity of driven cellular automata compared to linear combination of autonomous CA, with changing intensity of driving signal p(σ=1)∈[0,1].

As a case study we analyzed the complexity of hybrid elementary CA that execute two alternative rules based on the input signal. Figure 9 shows the variation in complexity of hybrid CAs with the driver mix p(σ=1) changing in [0,1]. For p=0 and p=1 the machine is regular elementary CA. We plotted the deviation of complexity from a linear mix, so-called synergy, as *p* is varied. Creating hybrid CAs using high and low complexity constituents results in drop in overall complexity (i.e., non-synergistic mix). The drop happens at even small mixing around the higher complexity CA. This compares to synergistic case when both constituents are of high complexity.

### 6.4. Susceptibility

Let $M^{A},M^{B}$ be two computrons operating on the same state space. We provide a measure of topological similarity between them by comparing their bendings of the configuration space. The bending ratio τ(s,q) was defined in Equation (46). The similarity of two computrons is given as:

(49) $$\Phi \left(M^{A},M^{B}\right)=\sum_{s,q\in S\times S}\left|\tau_{A}\left(s,q\right)-\tau_{B}\left(s,q\right)\right|$$

Equipped with a measure for similarity of two computrons, we can now analyze distortions in a machine upon small structural changes. As an application, we investigate distortion of a computron due to noise which is given as:

(50) $$\xi \left(\mu \right)=\Phi \left(M_{p_{\mu }=\mu },M_{p_{\mu }=0}\right)$$

**Figure 10:**
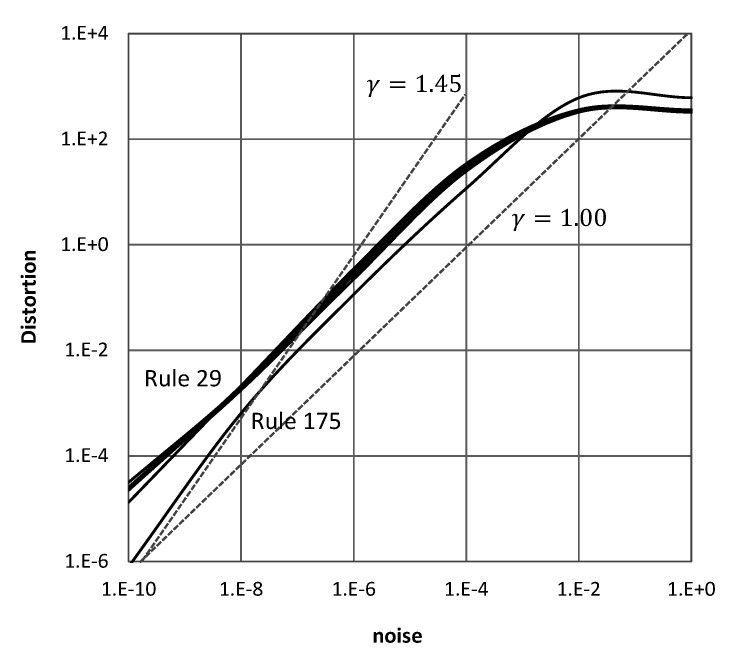
Topological distortion to driven computron due to noise. Distortion vs noise levels are plotted in log-log scale. Curves correspond to different levels of driver intensity which results in varying the machine’s behavior from Rule 29 to Rule 175 gradually.

Figure 10 shows noise distortion curves on log-log scale for a computron executing Rule 29.175 with varying levels of driver intensity. The machine exhibits linear noise distortion curves with saturation at high noise levels when it is driven in region Rule 29 towards Rule 175. However, as it gets closer to Rule 175, noise distortion become exponential with power γ≈1.45. This characterization is consistent with the sudden change in complexity around Rule 175 as was provided in Figure 9.

## 7. Discussion

This paper is an investigation into dynamics and complexity of a computation machine, computron, which is equivalent to a finite-state machine and generalization of a cellular automata. We embedded the computron into a metric space that captures similarity of configurations as patterns on the generating graph of computron. We analyzed the chaoticity and complexity of computron in this metric space. Our approach to complexity was basin-centric rather than path-centric. We also expanded the framework into non-autonomous machines with external drivers which led to Markov chain formalism. We applied our theory to hybrid elementary cellular automata with noise. Case studies showed that machines with high complexity are more susceptible to externalities, such as mixing and noise.

Our framework of conceptualizing automata as computrons in diversity space opens up a series of avenues for future research, including the study of robustness and complexity of various generating graphs (e.g., hierarchical) or connector families (e.g., reversible, linear). We can also investigate evolutionary dynamics of a computational machine by *‘growing’* (attaching cells to an existing machine) and *‘mutating‘* (altering generating graph or the connectors) computrons.

## Abbreviations

The following abbreviations are used in this manuscript:

FSA	finite-state automata
CA	cellular automata
DiD	diversity-induced distance

## Appendix A. Proof of Lemmas

**Lemma** **A1.**
*For any finite-state automaton with $2^{N}$ states there is an equivalent computron of N cells plus the input cell.*

Two machines $M=[S^{M},s_{0},\Sigma ,\Gamma ,T^{M},U^{M}]$ and $\Omega =[S^{\Omega },s_{0},\Sigma ,\Gamma ,T^{\Omega },U^{\Omega }]$ are said to be *equivalent* if there exists a one-to-one mapping between the states $\mu :S^{M}↔S^{\Omega }$, and for all $s\in S^{M}$ and σ∈Σ:

(A1) $$\begin{array}{cc} \mu (T^{M}\left(s,\sigma \right)) & =T^{\Omega }\left(\mu \left(s\right),\sigma \right)\\ U^{M}\left(s,\sigma \right) & =U^{\Omega }\left(\mu \left(s\right),\sigma \right) \end{array}$$

A generating graph G(V,E) is said to be *fully connected* if $\forall v_{i},v_{j}\in V,\exists e_{ji}\in E$. A state of a computron is the configuration of its cell states $s^{\Omega }=\left[\omega_{0}\omega_{1}...\omega_{N-1}\right],\omega_{i}\in \left\{0,1\right\}$ with *base indexing* defined as $k\left(s^{\Omega }\right)=\sum_{i=0}^{N-1}2^{\omega_{i}}$, where $N=|V_{G}|$ is the number of vertices of the graph. following 3 propositions construct the proof for the Lemma.

**Proposition** **A1.**
*For any computron Ω with an arbitrary generating graph G there exists an equivalent computron $\Omega^{*}$ with a fully connected generating graph $G^{*}$ with same number of vertices.*

We pair up cells of Ω and $\Omega^{*}$ as $v_{i}↔v_{i}^{*}$ and we use base indexing to enumerate configurations $s^{\Omega }$ and $s^{\Omega^{*}}$. This establishes the one-to-one mapping between the two configuration spaces $\mu :S^{\Omega }↔S^{\Omega^{*}}$. Then we construct the connectors $\left[C^{*}\right]$ of $\Omega^{*}$ as follows: Let $\delta_{i}=\left[v_{i_{0}}...v_{i_{n(i)}}\right]$ be the neighbors of cell $v_{i}$, and let $\delta_{i}^{{}^{\prime }}$ be the rest of the cells (i.e., not-connected to $v_{i}$.), then we set the loop-up tables of connectors $C_{i}^{*}$ for each cell as follows:

(A2) $$C_{i}^{*}\left(\left[\delta_{i},\delta_{i}^{{}^{\prime }}\right],\sigma \right)=C_{i}\left(\left[\delta_{i}\right],\sigma \right)$$

With output maps also set identical, $U^{*}\left(s,\sigma \right)=U\left(s,\sigma \right)$, then the implied Transition Maps $T^{\Omega }\left(s\right)$ and $T^{\Omega^{*}}\left(s\right)$ satisfy Equation (A1). Therefore, $\Omega \equiv \Omega^{*}$.

**Proposition** **A2.**
*There is one-to-one correspondence between finite-state automata M of $2^{N}$ states with binary input and fully connected computrons $\Omega^{*}$ with N+1 cells, including the input cell.*

Let $U_{\Omega^{*}}$ denote set of all fully connected computrons with N+1 cells (including the input cell).

(A3) $$|U_{\Omega^{*}}|={\left(2^{N}\right)}^{\left(2^{N}\right)}\times {\left(2^{N}\right)}^{\left(2^{N}\right)}$$

Each cell of $\Omega^{*}$ (other than the special input cell) has N+1 inputs from other cells and itself (fully connected) with $2^{N+1}$ input configurations. With each input configuration having a binary output set by the connector, the number of different connector types are $2^{\left(2^{N+1}\right)}$. Each cell, other than the input cell, can have any of the connector types (input cell has a unique connector that solely acts as interface to external input). Therefore, total number of different computrons with fully connected generating graph is ${\left(2^{2^{N+1}}\right)}^{N}={\left(2^{N}\right)}^{\left(2^{N}\right)}\times {\left(2^{N}\right)}^{\left(2^{N}\right)}$.

Let *M* be a finite-state automaton with $2^{N}$ states, and let $U_{M}$ denote set of all such machines. Since each state can go to any state based on the binary external input, we have:

(A4) $$\begin{array}{cc} |U_{M}| & ={\left(2^{N}\right)}^{\left(2^{N}\right)}\times {\left(2^{N}\right)}^{\left(2^{N}\right)}\\ \; & =|U_{\Omega^{*}}| \end{array}$$

Base indexing provides the one-to-one map between the configurations of $\Omega^{*}$ and (excluding the input cell) and the states of *M*. From Proposition A1, for each computron there is an equivalent fully connected computron. From A2, there is a one-to-one mapping between finite-state automata of $2^{N}$ states with binary input and a fully connected computron of N+1 cells, including the input cell. Therefore, for any finite-state automata there exists an equivalent computron.

**Lemma** **A2** (Diversity measure)**.**
*Diversity is a sub-additive measure on Q.*

Monotonicity:

(A5) $$A\tilde{\subset }B\Rightarrow {∥A∥}_{w}<{∥B∥}_{w}$$

Let A={a,b,c},B={a,b,c,x} with corresponding weights $\left\{w_{a}^{A},w_{b}^{A},w_{c}^{A}\right\}$ and $\left\{w_{a}^{B},w_{b}^{B},w_{c}^{B},w_{x}^{B}\right\}$, respectively, satisfying subset definition given in Equation (17). Let $\vec{A^{*}}=\left[bac\right]$ be the optimal ordering of A-tuple that maximizes $\sup_{\vec{A}}{∥\vec{A}∥}_{w}$, and construct $\vec{B^{*}}=\left[bacx\right]$.

(A6) $$\begin{array}{ccccc} ∥\vec{A^{*}}∥ & =w_{a}^{A}+ & w_{b}^{A}\rho_{ab}+ & w_{c}^{A}\rho_{cb}\rho_{ca}\\ ∥\vec{B^{*}}∥ & =w_{a}^{B}+ & w_{b}^{B}\rho_{ab}+ & w_{c}^{B}\rho_{cb}\rho_{ca}+ & w_{x}^{B}\rho_{xb}\rho_{xa}\rho_{xc} \end{array}$$

Therefore, ${∥A∥}_{w}=∥\vec{A^{*}}{∥}_{w}<∥\vec{B^{*}}{∥}_{w}<\sup_{\vec{B}}∥\vec{B}{∥}_{w}={∥B∥}_{w}□$

Sub-additivity:

(A7) $$∥A_{0}\tilde{\cup }A_{1}{∥}_{w}\leq ∥A_{0}{∥}_{w}+{∥A_{1}∥}_{w}$$

Let A={a,x}, B={b,x} and $C=A\tilde{\cup }B=\left\{a,b,x\right\}$ with corresponding weights $\left\{w_{a}^{A},w_{x}^{A}\right\}$, $\left\{w_{b}^{B},w_{x}^{B}\right\}$, and $\left\{w_{a}^{A},w_{b}^{B},w_{x}^{A}+w_{x}^{B}\right\}$, respectively. Let $\vec{C^{*}}=\left[bxa\right]$ be the optimal ordering of C-tuple that maximizes $\sup_{\vec{C}}{∥\vec{C}∥}_{w}$. Construct $\vec{A^{*}}=\left[xa\right]$ and $\vec{B^{*}}=\left[bx\right]$ as lifting from $\vec{C^{*}}$ with same ordering within. Then:

$$\begin{array}{cccc} ∥\vec{C^{*}}{∥}_{w} & =(w_{b}^{B}) & +\left(w_{x}^{A}+w_{x}^{B}\right)\rho_{xb} & +\left(w_{a}^{A}\right)\rho_{ab}\rho_{ax}\\ ∥\vec{A^{*}}{∥}_{w} & = & +(w_{x}^{A}) & +\left(w_{a}^{A}\right)\rho_{ax}\\ ∥\vec{B^{*}}{∥}_{w} & =(w_{b}^{B}) & +\left(w_{x}^{B}\right)\rho_{xb} \end{array}$$

Since ρ∈[0,1], ${∥C∥}_{w}=∥\vec{C^{*}}{∥}_{w}\leq ∥\vec{A^{*}}{∥}_{w}+∥\vec{B^{*}}{∥}_{w}\leq {∥A∥}_{w}+{∥B∥}_{w}$.

**Lemma** **A3** (Diversity-Induced Distance)**.**
*‘DiD’ defined as $\varphi \left(A,B\right)=\frac{∥A\tilde{▵}{B∥}_{w}}{\left|Q\right|}$ is a metric of $Q^{*}$*

Let $A,B,C\in Q^{*}$ and diversity-induced distance $\varphi \left(A,B\right)=\frac{∥A\tilde{▵}{B∥}_{w}}{\left|Q\right|}$, then from definitions φ(A,A)=0, and φ(A,B)=φ(B,A)≥0. We need to show the triangle-inequality property to establish DiD as a metric: φ(A,B)≤φ(A,C)+φ(C,B). First, we will show that $A\tilde{▵}B\subseteq \left(A\tilde{▵}C\right)\tilde{\cup }\left(C\tilde{▵}B\right)$. Let A={a,x,y,p}, B={b,x,x,p}, and C={c,y,z,p} with corresponding weights:

$A\tilde{▵}C$	$C\tilde{▵}B$	$A\tilde{▵}B$	$\left(A\tilde{▵}C\right)\tilde{\cup }\left(C\tilde{▵}B\right)$
$w_{a}$		$w_{a}$	$w_{a}$
	$w_{b}$	$w_{b}$	$w_{b}$
$w_{c}$	$w_{c}$		$w_{c}$
$w_{x}^{A}$	$w_{x}^{B}$	$|w_{x}^{A}-w_{x}^{B}|$	$\left(w_{x}^{A}+w_{x}^{B}\right)$
$|w_{y}^{A}-w_{y}^{C}|$	$w_{y}^{C}$	$w_{y}^{A}$	$|w_{y}^{A}-w_{y}^{C}|+w_{y}^{C}$
$w_{z}^{C}$	$|w_{z}^{C}-w_{z}^{B}|$	$w_{z}^{B}$	$w_{z}^{C}+\left|w_{z}^{C}-w_{z}^{B}\right|$
$|w_{p}^{A}-w_{p}^{C}|$	$|w_{p}^{C}-w_{p}^{B}|$	$|w_{p}^{A}-w_{p}^{B}|$	$|w_{p}^{A}-w_{p}^{C}|$
			$+|w_{p}^{C}-w_{p}^{B}|$

For each row, weight for $A\tilde{▵}B$ is less than or equal to the weight for $\left(A\tilde{▵}C\right)\tilde{\cup }\left(C\tilde{▵}B\right)$. Therefore, $A\tilde{▵}B\subseteq \left(A\tilde{▵}C\right)\tilde{\cup }\left(C\tilde{▵}B\right)$. Then the rest follows from Lemma A2:

(A8) $$\begin{array}{cc} A\tilde{▵}B & \subseteq \left(A\tilde{▵}C\right)\tilde{\cup }\left(C\tilde{▵}B\right)\\ ∥A\tilde{▵}{B∥}_{w} & \leq ∥\left(A\tilde{▵}C\right)\tilde{\cup }\left(C\tilde{▵}B\right){∥}_{w}\\ \; & \leq ∥A\tilde{▵}{C∥}_{w}+{∥C\tilde{▵}B∥}_{w} \end{array}$$

**Lemma** **A4** Maximal entanglement)**.** (*Let $P=\{B_{0},B_{1}\cdots \}$ be a partitioning of Q (i.e., $\cup_{i}B_{i}=Q$, and $B_{i}\cap B_{j}=\emptyset ,i\neq j$) with $P^{0}=\left\{Q\right\}$ and $P^{\infty }=\left\{\left\{x_{0}\right\},\left\{x_{1}\right\},\cdots \right\},\forall x_{i}\in Q$, then:*

(A9) $$∥P^{0}∥=∥P^{\infty }∥\geq ∥P∥$$

The proof is based on a combinatorics argument. Entanglement of $P^{0}$ is calculated by shuffling all the elements of universe *Q* within a single set, whereas entanglement of $P^{\infty }$ is calculated by shuffling sets of all single elements, to find the supremum.

(A10) $$\begin{array}{cc} ∥P^{0}∥=\underset{\vec{P}}{\sup}D\left(\vec{P}\right) & =∥Q∥=\underset{\vec{Q}}{\sup}d\left(\vec{Q}\right)\\ ∥P^{\infty }∥=\underset{\vec{P}}{\sup}D\left(\vec{P}\right) & =∥\left\{x_{i}\right\}∥+∥\left\{x_{j}\right\}|\left\{x_{i}\right\}∥+\cdots \\ \; & =d\left(x_{i}\right)+d\left(x_{j}|x_{i}\right)+\cdots \\ \; & =\underset{\vec{Q}}{\sup}d\left(\vec{Q}\right) \end{array}$$

Therefore, $∥P^{0}∥=∥P^{\infty }∥$. For any other partition P, entanglement is calculated by shuffling the set blocks of the partition together with shuffling the elements within each set block. Therefore, its supremum is less than or equal to that of $P^{0}$ which seeks the supremum through all possible orderings. As a simple case consider Q={a,b,c}. Let $P^{0}=\left\{\left\{a,b,c\right\}\right\},P^{\infty }=\left\{\left\{a\right\},\left\{b\right\},\left\{c\right\}\right\}$, and $P^{1}=\left\{\left\{a,b\right\},\left\{c\right\}\right\}$

$∥P^{0}∥$	$∥P^{\infty }∥$	$∥P^{1}∥$
$\sup_{A}d\left(.\right)$	$\sup_{B}d\left(.\right)$	$\sup_{C}d\left(.\right)$
A	B	C
abc	abc	abc
acb	acb	
bac	bac	bac
bca	bca	
cab	cab	cab
cba	cba	cba

Therefore, $∥P^{0}∥=∥P^{\infty }∥\geq ∥P∥$.

**Lemma** **A5** (Basin partition)**.**
*The set of all basins of an autonomous computron is a partition of its configuration space, denoted as $P_{M}=\left\{B_{0},B_{1},\cdots \right\}$, with the corresponding attractors $A_{M}=\left\{\vec{A_{0}},\vec{A_{1}},\cdots \right\}$.*

A partition of *S* is $P=\{B_{0},B_{1},\cdots \}$, where $B_{i}\subset S$, $\cup_{i}B_{i}=S$ and $B_{i}\cap B_{j}=\emptyset ,i\neq j$. So, we need to show that each state belongs to some basin and no state belongs to more than one basin. Due to finite-state nature of the computron this is trivial. Let $P_{n}\left(s_{0}\right)=\left\{s_{0},s_{1},..s_{n}\right\}$ be the n-step forward path of state $s_{0}$. Since |S|<∞,∃k≤|S| such that $s_{k+1}\in P_{k}\left(s_{0}\right)$ and $s_{i+1}\notin P_{i}\left(s_{0}\right),\forall i<k$. Denote $s_{k+1}=s_{q}\left(q\leq k\right)$. Then, $\vec{A}=\left[s_{q},s_{q+1}...s_{k}\right]$ is an attractor of the computron, as per definition, and $s_{0}\in B\left(\vec{A}\right)$. Therefore, each state is an element of some basin.

Now we show that no state belongs to more than one basin. Assume $s\in B({\vec{A}}_{a})$ and $s\in B({\vec{A}}_{b})$ with ${\vec{A}}_{a}=\left[s_{q_{a}}\cdots s_{k_{a}}\right]$ and ${\vec{A}}_{b}=\left[s_{q_{b}}\cdots s_{k_{b}}\right]$. If $k_{b}<k_{a}$ then $\exists i=k_{b}∋s_{i+1}\in P_{i}\left(s\right)$ which means ${\vec{A}}_{b}$ is not an attractor as it violates attractor definition, and similarly if $k_{a}<k_{b}$ then ${\vec{B}}_{b}$ is not an attractor. Therefore $k_{a}=k_{b}$ and ${\vec{A}}_{a}={\vec{A}}_{b}$.

**Lemma** **A6** (Minimal complexity)**.**
*Absorbing and idle computrons have the least complexity (nil) compared to any other computron.*

(A11) $$\Psi_{M^{0}}=\Psi_{M^{\infty }}=0\leq \Psi_{M}$$

Absorbing computron $M^{0}$ has a single fixed-point attractor which all states iterate into therefore its basin partitioning is $P_{M^{0}}=\left\{S\right\}=P^{0}$, and idle computron $M^{\infty }$ each state is mapped to itself, therefore its basin partitioning is $P_{M^{\infty }}=\left\{\left\{s_{0}\right\},\left\{s_{1}\right\}\cdots \right\}=P^{\infty }$. Hence from definition of complexity, $\Psi_{M^{0}}=\Psi_{M^{\infty }}=0\leq \Psi_{M}$.

**Lemma** **A7** (Coloring)**.** *Let $P_{a},P_{b}$ two random initial distributions with non-zero entries for each state, and let $\pi_{a}$ and $\pi_{b}$ be the corresponding steady-state distributions in an asynchronous computron. Define, the* ‘gray color’ *of a state c(s) as:*

(A12) $$c\left(s\right)=\left\{\begin{array}{cc} \pi_{b}\left(s\right)/\pi_{a}\left(s\right) & \mathrm{if}\;\pi_{a}\left(s\right)>0\\ 0 & \mathrm{else} \end{array}\right.$$

Two states will, almost always, have the same shade of gray if and only if they are elements of same recurrent class, provided all classes of the computron are *’distinguishable’*; i.e., for two random non-zero initial distributions, $c\left(s\right)=c\left(q\right)>0\Leftrightarrow s,q\in C_{i}$, almost always.

Let $C_{M}=\left\{C_{1},C_{2},\cdots ,C_{K}\right\}$ be the recurrent classes of a computron, and $\pi_{M}=\left\{\pi_{1},\pi_{2},\cdots \pi_{K},\right\}$ the corresponding steady-state distributions of the class. Then:

(A13) $$\pi_{a}=\sum_{k=1}^{K}\chi_{k}\left(p_{a}\right)\pi_{k},\;\pi_{b}=\sum_{k=1}^{K}\chi_{k}\left(p_{b}\right)\pi_{k}$$

First we show, $s,q\in C_{m}\Rightarrow c\left(s\right)=c\left(q\right)$. By definition recurrent (see Terminology) classes are non-overlapping. If $s,q\in C_{m}$, then:

(A14) $$\begin{array}{cc} \pi_{a}\left(s\right)=\chi_{m}\left(p_{a}\right)\pi_{m}\left(s\right), & \;\pi_{a}\left(q\right)=\chi_{m}\left(p_{q}\right)\pi_{m}\left(q\right)\\ \pi_{b}\left(s\right)=\chi_{m}\left(P_{b}\right)\pi_{m}\left(s\right), & \;\pi_{b}\left(q\right)=\chi_{m}\left(P_{b}\right)\pi_{m}\left(q\right) \end{array}$$

Therefore,

(A15) $$c\left(s\right)=\pi_{a}\left(s\right)/\pi_{b}\left(s\right)=\frac{\chi_{m}\left(p_{a}\right)}{\chi_{m}\left(p_{b}\right)}=\pi_{a}\left(q\right)/\pi_{b}\left(q\right)=c\left(q\right)$$

We define *‘distinguishable’* as follows: Let ${\vec{W}}_{x\to y}=x_{0}x_{1}...x_{k}$ be a *walk* state $s_{x}$ to state $s_{y}$. We define the walk probability as $p\left({\vec{W}}_{x\to y}\right)=\prod_{i=0}^{k-1}P_{x_{i}x_{i+1}}$. Let ${\tilde{W}}_{x\to y}$ denote set of all different walks from state $s_{x}$ to state $s_{y}$. We define reach probability from $s_{x}$ to $s_{y}$ as $p_{x\to y}=\sum_{\vec{W}\in \tilde{W}}p\left({\vec{W}}_{x\to y}\right)$. We define reach probability from a state $s_{x}$ to an attractor *A* as $p_{x\to A}=\sum_{s_{y}\in A}p_{x\to y}$, where the walk from $s_{y}\in A$ there does not have any other element of *A*. Two recurrent classes *A* and *B* are said to be *‘equidistant’* with respect to state $s_{x}$, if $p_{x\to A}=p_{x\to B}$. Two recurrent classes *A* and *B* are said to be not *‘distinguishable’* if they are equidistant with respect to all states in *S*. Define the |S|×|C| matrix $P^{C}$ as follows:

(A16) $$P^{C}=\left[\begin{array}{cccc} p_{0\to C_{1}} & p_{0\to C_{2}} & \cdots & p_{0\to C_{K}}\\ p_{1\to C_{1}} & p_{1\to C_{2}} & \cdots & p_{1\to C_{K}}\\ \vdots & \; \end{array}\right]$$

If any two columns of $P^{C}$ are equal then the corresponding recurrent classes are indistinguishable. Let $\chi \left(P_{a}\right)=\left[\chi_{1}\left(P_{a}\right)\cdots \chi_{K}\left(P_{a}\right)\right]$, then:

(A17) $$\chi \left(p_{a}\right)=p_{a}P^{C}$$

Now, we show that almost always, for any two recurrent classes $C_{m},C_{n}$ that are *’distinguishable’*, $s\in C_{m},q\in C_{n}\Rightarrow c\left(s\right)\neq c\left(q\right)$. Assume the contrary:

(A18) $$\begin{array}{cc} c(s)=c(q) & \Rightarrow \frac{\chi_{m}\left(p_{a}\right)}{\chi_{m}\left(p_{b}\right)}=\frac{\chi_{n}\left(p_{a}\right)}{\chi_{n}\left(p_{b}\right)}\\ \; & \Rightarrow \frac{p_{a}.p^{C}\left[m\right]}{p_{a}.P^{C}\left[n\right]}=\frac{p_{b}.P^{C}\left[m\right]}{p_{b}.p^{C}\left[n\right]} \end{array}$$

The set of distinct initial distributions pairs $p_{a},p_{b}$ that are solution to Equation (A18) represent a plane in ${ℜ}^{|}S|$ which may or may not be a probability distribution. The ratio of size of this set to size of all possible initial distribution pairs is $\frac{O(|S|-1)}{O(|S|)}\approx 0$, hence contrary assumption leads to contradiction almost always.

**Lemma** **A8** (Ergodic bases)**.**
*Recurrent classes form a non-overlapping spanning set (basis) for the impact sets of all states. Let $C_{M}=\left\{C_{1},C_{2},\cdots ,C_{K}\right\}$ be the set of recurrent classes of a computron and $\chi_{s}=\left\{\chi_{1}\left(s\right),\chi_{2}\left(s\right),\cdots \chi_{K}\left(s\right)\right\}$ the ergodic decomposition of impulse response $\pi_{s}$:*

(A19) $$\chi_{k}\left(s\right)=\sum_{q\in C_{k}}\pi_{s}\left(q\right)$$

*then:*

(A20) $$\sum_{k}\chi_{k}\left(s\right)=1,\;\mathrm{with}\;\chi_{k}\left(s\right)\geq 0,\forall s\in S$$

(A21) $$\begin{array}{cc} \chi (p_{a}) & =P_{a}P^{C}\\ \chi_{k}\left(s\right)=P_{s}^{\delta }P^{C}\left[k\right] & =P^{C}\left[s\right]\left[k\right]=p_{s\to C_{k}} \end{array}$$

(A22) $$\sum_{k}\chi_{k}\left(s\right)=\sum_{k}p_{s\to C_{k}}=1$$

**Lemma** **A9** Fuzzy Partition)**.** (*The set of all basins of attraction of a driven computron is a fuzzy partition of its configuration space, denoted as ${\tilde{P}}_{M}=\left\{\tilde{B}\left(C_{1}\right),\tilde{B}\left(C_{2}\right),\cdots ,\tilde{B}\left(C_{K}\right)\right\}$.*

Basins of attraction for an asynchronous computron is a weighted-set $\tilde{B}\left(C_{k}\right)$:

(A23) $$\tilde{B}\left(C_{k}\right)=\left\{s:\chi_{k}\left(s\right)>0,w_{\tilde{B}}\left(s\right)=\chi_{k}\left(s\right)\right\}$$

A fuzzy partition of *S* is the set of weighted sets $\tilde{P}=\left\{{\tilde{B}}_{1},{\tilde{B}}_{2},..{\tilde{B}}_{K}\right\}$, where $\cup_{i}{\tilde{B}}_{i}=S$, and $\sum_{k}w_{{\tilde{B}}_{k}}\left(s\right)=1,\forall s\in S$. The proof follows directly from definition of basin, Equation (A23) and Lemma (A8).
